# Cyclic PaO_2_ oscillations assessed in the renal microcirculation: correlation with tidal volume in a porcine model of lung lavage

**DOI:** 10.1186/s12871-017-0382-7

**Published:** 2017-07-11

**Authors:** Rainer Thomas, Christian Möllmann, Alexander Ziebart, Tanghua Liu, Matthias David, Erik K. Hartmann

**Affiliations:** Department of Anesthesiology, Medical Center of the Johannes Gutenberg-University, Mainz, Germany

**Keywords:** Ards, Cyclic recruitment, Lung injury, Organ crosstalk, Renal failure

## Abstract

**Background:**

Oscillations of the arterial partial pressure of oxygen induced by varying shunt fractions occur during cyclic alveolar recruitment within the injured lung. Recently, these were proposed as a pathomechanism that may be relevant for remote organ injury following acute respiratory distress syndrome. This study examines the transmission of oxygen oscillations to the renal tissue and their tidal volume dependency.

**Methods:**

Lung injury was induced by repetitive bronchoalveolar lavage in eight anaesthetized pigs. Cyclic alveolar recruitment was provoked by high tidal volume ventilation. Oscillations of the arterial partial pressure of oxygen were measured in real-time in the macrocirculation by multi-frequency phase fluorimetry and in the renal microcirculation by combined white-light spectrometry and laser-Doppler flowmetry during tidal volume down-titration.

**Results:**

Significant respiratory-dependent oxygen oscillations were detected in the macrocirculation and transmitted to the renal microcirculation in a substantial extent. The amplitudes of these oscillations significantly correlate to the applied tidal volume and are minimized during down-titration.

**Conclusions:**

In a porcine model oscillations of the arterial partial pressure of oxygen are induced by cyclic alveolar recruitment and transmitted to the renal microcirculation in a tidal volume-dependent fashion. They might play a role in organ crosstalk and remote organ damage following lung injury.

## Background

Adverse effects of remote organ crosstalk is a common and severe problem in intensive care patients and a novel field of research [1]. Organ dysfunctions, especially acute respiratory distress syndrome (ARDS) and acute kidney injury, can be caused by the same external etiology, but also trigger each other. The combination of both is linked to prolonged intensive care stays and increased mortality [2–4]. The underlying pathways are not fully understood, but are likely linked to increased inflammation [5]. Respiratory cycle-dependent recruitment and derecruitment of lung parenchyma (cRD), defined as atelectotrauma, plays an important role in ventilator-induced lung injury by applying shearing forces to lung tissue, which lead to pulmonary and/or systemic inflammation [6]. In addition to pulmonary injury, cRD generates varying shunt fractions within the respiratory cycle that lead to ultrafast oscillations of the arterial oxygen partial pressure (PaO_2_) and can be detected in macrocirculation, microcirculation, and even in brain tissue [7–10]. A novel technique based on multi-frequency phase fluorimetry (MFPF) can be used to detect PaO_2_ oscillations in real-time. For this purpose a ruthenium-covered probe is inserted into the arterial circulation. Currently, an advanced version of this technique allows continuous real-time measurements [8, 11].

Several experimental studies recently addressed the influence of various respiratory patterns or modes on the occurrence and extent of these oscillations [12–15]. Preclinical data suggest that the occurrence of intermitting oxygen oscillations per se might represent a trigger for remote organ failure following lung injury [6, 16–19]. The role of the tidal volume as driving force for PaO_2_ oscillations, their transmission to various distant organ microcirculatory sites and particularly the kidney is yet unknown.

We hypothesized that oscillations of PaO_2_ that are induced by cRD are (I) transmitted to the renal tissue and change oxygen saturation at the capillary level, and that (II) the magnitude of this effect depends on the respiratory tidal volume. This transmission may be a possible pathway of the injured lung directly affecting renal integrity.

## Methods

### Preparation

The protocol was approved by the State and Institutional Animal Care Committee (Rhineland-Palatinate, Germany, ID G12–1-059). Pilot experiments were performed to develop the protocol. Eight pigs (27–28 kg) were acquired through a local farmer and delivered to the laboratory following sedation (Azaperone, Ketamine). Induction of anesthesia was accomplished with fentanyl (4 μg/kg) and propofol (5–6 mg/kg) via an ear vein cannula. Anesthesia was maintained by continuous infusion of propofol (5–10 mg/kg/h) and fentanyl (0.05–0.1 mg/kg/h). Atracurium (0.5 mg/kg) was administered intravenously only to facilitate endotracheal intubation. Basic monitoring included pulse oximetry (Masimo Radical 7, Irvine California, USA) and spirometric monitoring (S/5, GE-Datex-Ohmeda, Chalfont St. Giles, United Kingdom). The animals were ventilated using a volume-controlled mode (AVEA, Carefusion, San Diego, USA) with a tidal volume of 7 ml/kg, positive endexpiratory pressure (PEEP) of 5 mbar, fraction of inspired oxygen (FiO_2_) of 0.4 and respiratory rate oriented on the end-tidal CO_2_. Femoral vascular access was achieved using Seldinger’s technique after ultrasound-guided puncture for placement of a central venous line, an arterial introducer for ultrafast PaO_2_ measurement, and a pulse contour cardiac output catheter (PiCCO, Pulsion Medical, Munich, Germany). The data from all devices was monitored and stored continuously. Balanced electrolyte fluid was administered at an infusion rate of 5–15 ml/kg/h. To compensate fluid deficiency and blood loss during the instrumentation 500 ml of hydroxyethyl starch 130/0.4 were administered over 2 h. If necessary, noradrenaline was administered to maintain a mean arterial pressure above 60 mmHg.

### Ultrafast PaO_2_ measurement in the macrocirculation using multi-frequency phase fluorimetry

A ruthenium-covered probe was inserted via the femoral artery introducer (Foxy-AL300, Ocean Optics, USA) as previously reported [13, 14]. The tip of the probe is covered with ruthenium-based fluorescent molecules, whose fluorescence is quenched by the presence of molecular oxygen. A fiber optic bundle sends blue LED light to the sensor and the resulting fluorescence is relayed back through the bundle to the detector (NeoFox, Ocean Optics, USA). The decay profile of the fluorescent molecules changes in the presence of molecular oxygen, and arterial PaO_2_ is calculated automatically by the dedicated software with a time resolution of up to 10 Hz.

### Ultrafast renal oxygen saturation (SrO_2_) measurement

Access to the kidney was achieved surgically and the probe was placed manually on the surface of the kidney at the height of the hilus. SrO_2_ was measured by means of laser Doppler flowmetry and white light spectroscopy (O2C, LEA Medizintechnik, Giessen, Germany) as previously reported by Klein et al. for assessment of peripheral and cerebral microcirculation [8, 10]. The light absorption of hemoglobin varies depending on the oxygenation saturation. White light is emitted by the probe, and the spectrum of measured light allows the calculation of the tissue saturation. The amplitude of measured light allows the calculation of hemoglobin amount. Capillary and small venous vessels contain the majority of tissue hemoglobin and therefore contribute the most to the measurement.

### Study protocol

ARDS was induced by surfactant washout using repetitive bronchoalveolar lavages. The endotracheal tube was closed during inspiration. 30 ml/kg of warmed balanced saline solution were instilled by gravity and immediately removed. This procedure was repeated until the PaO_2_/FiO_2_ ratio remained less than 200 mmHg over 30 min. To provoke considerable cRD and PaO_2_ oscillations, a dedicated algorithm that excludes the occurrence of relevant oscillations under healthy conditions and an aggressive respiratory regime was used: tidal volume of 25–30 ml/kg, respiratory rate of 5–7 per minute, zero PEEP, FiO_2_ 1.0, and inspiratory to expiratory ratio of 1:4 [13, 14]. Aortal PaO_2_, SrO_2_ and hemodynamic and respiratory data were continuously monitored in a time-synchronized manner. The tidal volume was reduced by decrements of 5 ml/kg until the PaO_2_ oscillations vanished. Each tidal volume was maintained for 5 min to ensure adequate equilibrium and steady-state conditions. Samples for blood gas analysis were withdrawn at each step very slowly over several respiratory cycles. The experiment was ended in deep general anesthesia by injection of 200 mg of propofol and 40 mmol of potassium chloride.

### Statistics

Values are given as averages of individual contributors and mean standard deviation. Statistical analysis was performed using Sigmaplot 12.5 (Systat Software Inc., San Jose, CA, USA). Oscillation amplitudes refer to peak to peak differences and were calculated semi-automatically via a previously described Fourier-analysis-routine using MathCad (Parametric Technology Corporation, Needham, MA, USA) [12]. To compare values before and after intervention, the Wilcoxon-test was used. To determine correlations, the Pearson-coefficient was used. *P*-values of less than 5% were considered to be statistically significant.

## Results

Hemodynamics and ventilation parameters remained stable during all measurements. The alveolar lavage created significant acute lung injury as demonstrated by the PaO_2_/FiO_2_ ratio dropping from 505 ± 49 to 202 ± 90 mmHg. Cardio-respiratory measurements during baseline assessment and cRD are summarized in Table 1.

**Table 1:** Measurements during baseline assessment and cRD with maximum tidal volume

Value	Unit	Baseline	cRD	*P*-value
CO	l/min	3.7 ± 0.6	3.8 ± 1.0	n.s.
MAP	mmHg	94 ± 16	84 ± 11	n.s.
RR	1/min	34 ± 4	6 ± 1	<0.01
PaO_2_/FiO_2_	mmHg	505 ± 49	205 ± 90	<0.01
P_plat_	mbar	14 ± 2.	32 ± 5	<0.01
PEEP	mbar	5 ± 1	0.1 ± 1	<0.01
V_t_	ml/kg	7.5 ± 0.3	27.7 ± 1.5	<0.01
pH	-	7.47 ± 0.05	7.35 ± 0.06	<0.01
pCO_2_	mmHg	44.58 ± 4.37	62.73 ± 7.61	<0.01

CO – cardiac output, MAP – mean arterial pressure, RR – respiratory rate, P_plat_ – plateau pressure, PEEP – positive end expiratory pressure, V_t_ –tidal volume, pH – blood pH via blood gas analysis, pCO_2_ – arterial CO_2_ pressure via blood gas analysis, n.s. – not significant

**Fig. 1:**
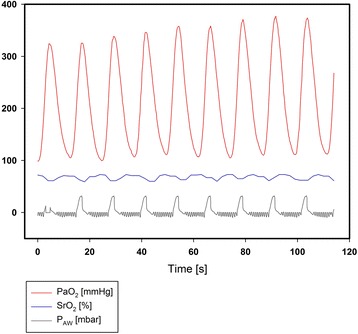
Exemplary real-time data of recruitment and derecruitment induced PaO_2_ oscillations, corresponding transmission to the renal microcirculation (oscillations of renal tissue saturation, SrO_2_) and correlation to the airway pressure (P_AW_), which indicates the respiratory-dependent character

**Fig. 2:**
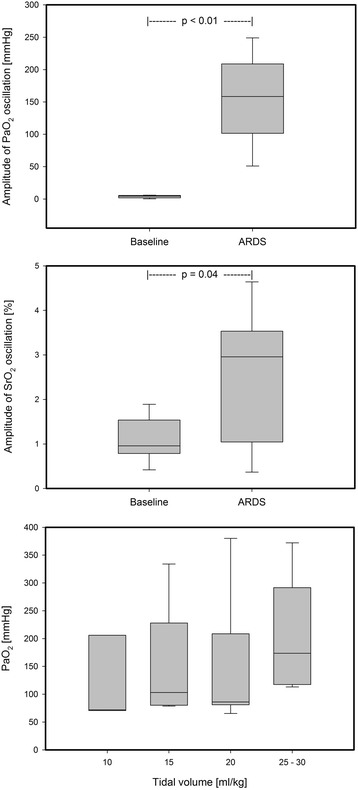
Amplitudes of PaO_2_ and SrO_2_ oscillations during cRD in comparison to the baseline values (upper graphs). PaO_2_measured by blood gas analysis during tidal volume down-titration (lower graph)

**Fig. 3:**
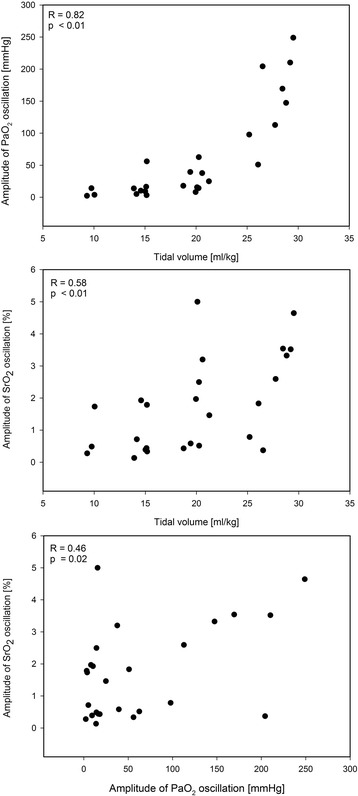
Pearson correlation of applied tidal volume and PaO_2_ or SrO_2_ oscillation amplitudes, Pearson correlation of PaO_2_ oscillation and SrO_2_ oscillation

During baseline conditions we neither recorded oscillations in PaO_2_ nor in SrO_2_. Application of the aggressive respirator setting in healthy conditions resulted in average oscillation of 22 mmHg without SrO_2_ oscillations. No inverse oscillations that can be attributed to perfusion alterations were found [20]. Following acute lung injury and cRD provocation, large PaO_2_ oscillation amplitudes of 155 ± 66 mmHg occurred. These were accompanied by corresponding SrO_2_ oscillations (2.6 ± 1.5%) as shown in Fig. 1. The PaO_2_ and SrO_2_oscillation amplitudes significantly differed from the baseline value (Fig. 2). The down-titration of the applied tidal volume immediately reduced the PaO_2_ and SrO_2_ oscillation amplitudes despite comparable absolute PaO_2_ (Figs. 2, 3). The corresponding airway driving pressures were 32 ± 4 mbar during initial setting and decreased during tidal volume titration (20 ml/kg: 29 ± 4, 15 ml/kg: 26 ± 4, 10 ml/kg: 23 ± 3 [mbar]). The extent of the PaO_2_ and SrO_2_ oscillations significantly correlated with the applied tidal volume (Pearson Coefficient *R* = 0.82 respectively *R* = 0.58, each *p* < 0.01; Fig. 3). Oscillation amplitudes of SrO_2_ and PaO_2_ as well correlated with each other (Pearson Coefficient *R* = 0.46, *p* = 0.02). Tidal volume reduction down to 10 ml/kg eliminated ongoing oxygen oscillations. The average of PaO_2_ measured via MFPF correlated with the corresponding value acquired via blood gas analysis (Pearson Coefficient *R* = 0.95, *p* < 0.01).

## Discussion

The present study reveals two key findings: systemic cRD-induced PaO_2_ oscillations are transmitted to the renal microcirculation and depend on the tidal volume. To our knowledge, this is the first study that shows a direct alteration of renal capillary oxygen saturation due to fast intratidal changes of shunt and PaO_2_ within the injured lung.

Dynamic lung stress leading to atelectrauma is proposed to be a major contributor of ventilator-induced damage within the injured lung [21, 22]. cRD exposed lung areas were shown to exert high amounts of inflammatory response [23]. Baumgardner et al. first reported the occurrence of large scale respiratory-dependent PaO_2_ oscillations caused by cRD in a rabbit model [12]. Interestingly, perfusion-related inverse oscillations that are not induced by cRD can also be provoked in healthy pigs [20]. An occurrence of small oscillation amplitudes (< 30 mmHg) during our aggressive respiratory setting even in healthy state is in line with previous reports [14]. The present proof of concept study did not directly assess the underlying mechanism of cRD and its correlation to PaO_2_ oscillations. This seems reasonable as it was sufficiently certified by several approaches [12, 14, 24, 25]. Due to the lack of clinical or bedside devices for detection of fast intratidal variations such as cRD, the detection of respiratory-dependent PaO_2_-oscillations was proposed as an indicator for indirect quantification of cRD [7].

cRD-induced variations of the PaO_2_ are transmitted into peripheral microcirculation and may lead to cyclically occurring episodes of hyper- or hypoxia [8]. Various cell types are compromised when exposed to cyclic oxygen variations both in a hyperoxic and hypoxic range: both ranges trigger were shown to be associated with inflammatory response [16, 17]. Furthermore, oxidative stress may play a role for oscillations leading to intermittent hyperoxia [16, 17]. Systemic PaO_2_ oscillations are also transmitted to the cerebral tissue [10]. Long-term exposure to PaO_2_ oscillations furthermore causes neuronal injury and inflammatory response within the brain, [6] which may represent a mechanism of deleterious organ crosstalk following single organ failure. Acute kidney injury is the most common organ failure in intensive care units [26]. In combination with ARDS it prolongs intensive care unit stays and significantly increases mortality, whereas ARDS itself is a risk factor for subsequent kidney injury [27]. Recent studies suggest several mechanisms for this organ crosstalk, e.g. an increase of systemic inflammatory markers, [4] changes in blood flow, edema, ischemia [28] as well as increased inflammation following antibiotics against bacterial sepsis [29]. However, the crosstalk between lung and kidney is not fully understood and respiratory-dependent SrO_2_ oscillations may add one more pathway of remote kidney injury that needs to be addressed in future research, because neither kidney damage nor function was directly assessed in this pilot study.

In previously published animal models that focused on occurrence and behavior of cRD-related PaO_2_ oscillations aggressive ventilator settings with highest tidal volumes were applied to provoke substantial cyclically recruitment of lung tissue [6, 8, 11–13, 23, 30]. The present study is the first one to focus on the tidal volume as a primary driving force for this phenomenon: a down-titration of the applied tidal volume to 10 ml/kg eliminates the PaO_2_ oscillations. This tidal volume is still considerably higher than those applied in ARDS patients in lung-protective therapy regimes. Computer tomographic clinical studies in ARDS patients document the occurrence of cRD even during lung protective tidal volume ventilation of 4–6 ml/kg [31–33]. Several aspects should be taken into account to understand these controversial results. Insensitivity of PaO_2_ oscillations in the detection of cRD and occurrence only in large areas of cyclically recruiting lung tissue cannot be fully excluded. However, Hartmann et al. showed that even small cyclically recruiting shunt fractions (lower than 5%) can cause detectable PaO_2_ oscillations [13]. The present study makes use of two advanced technical approaches at two locations within the bloodstream to depict cRD-related oxygen oscillations that correspond to tidal volume down-titration. Acute lung injury was induced by repetitive bronchoalveolar saline lavage, which is a commonly used model for rapid development of atelectasis and ARDS-like gas exchange impairment. The lavage model is particularly appropriate for studies concerning respiratory physiology or characteristics of atelectasis [34, 35]. The lavage model, though, is criticized for not appropriately reflecting the pattern of human ARDS. cRD is not an exclusive pattern of the lavage model and also occurs in different experimental conditions or models [36, 37] as well as in clinical ARDS studies. Tidal volume indeed seems to be the major determinant for cRD in this acute porcine model, which does not necessarily depict the clinical scenario of ARDS. The non-occurrence of PaO_2_ oscillations in lower tidal volume ranges in the present study cannot be generalized to clinical ARDS. In this context, combined laser-Doppler flowmetry and white light spectrometry [8] as well as rapid measurement of peripheral oxygen saturation [24] may allow for non-invasive, bedside detection of cRD-related PaO_2_ oscillations in lung injured patients. Formenti et al. recently developed a platinum-based sensor for ultrafast PaO_2_ measurement that overcomes the toxicity concerns of this study’s ruthenium-based device and may become available for measurement of real-time or respiratory-dependent PaO_2_ variations in humans [30, 38]. The analysis of PaO_2_ oscillations showed stronger *p*-values and better tidal volume correlation than the SrO_2_ oscillations. But the average PaO_2_ of 205 ± 90 mmHg during cRD limits the effect onto saturation due to the oxyhemoglobin dissociation curve. Additionally the effect may be limited by the autoregulation of renal blood flow.

Many factors have an impact on oxygen supply: ventilation, oxygen pressure of inspiratory gas, heart rate, cardiac output, perfusion distribution (shunt), metabolic status, oxyhemoglobin dissociation curve. For this study their impact was limited by the application of a standardized protocol and a pre-established algorithm for cRD provocation [13, 14]. Most parameters remained constant during measurements (e.g. metabolic status). The Fourier-transformation mathematically eliminated all impacts not synchronized to the ventilation frequency (e.g. heart rate). Cardiac output depends not only on heart rate, but also on intrathoracic pressure and its variation, which changes periodically at the ventilation frequency. Its impact cannot be eliminated by Fourier-transformation, but is unlikely to fully explain the alteration in SrO_2_ [8]. Hypercapnia in the ARDS group and a lower pH modify the oxygen dissociation curve. Their impact on the oscillations cannot be excluded in this study. Consistent with the present PaO_2_ (Fig. 2) the influence of the oxygen dissociation curve limits the extent of SrO_2_ oscillations. These showed a strict respiratory-dependent character and are in line with previous studies that assessed the transmission of PaO_2_ oscillation to the peripheral microcirculation [8, 24, 36]. This study served as a proof of concept in a porcine model, but results from long-term experiments or by means of clinically applicable techniques in patients are required to confirm the significance of the reported pathophysiological concept.

## Conclusion

The current study confirms two hypotheses in a porcine lung injury model. First, oscillations of PaO_2_ caused by high tidal volumes in ARDS pigs are transmitted to the renal microcirculation and can be measured at the venous capillary level. This suggests a possible pathway to the injury of organs other than the lung itself. Second, the amplitude of oscillations strongly depends on the tidal volume. This emphasizes the potential role of oxygen variations in deleterious organ crosstalk and highlights the importance of lung-protective ventilation to avoid cyclic alveolar recruitment.

## Abbreviations

ARDS: Acute respiratory distress syndrome
CO: Cardiac output
cRD: Cycle-dependent recruitment and derecruitment of lung parenchyma
F_i_O_2_: Fraction of inspired oxygen
MAP: Mean arterial pressure
MFPF: Multi-frequency phase fluorimetry
n.s.: Not significant
PaO_2_: Arterial oxygen partial pressure
P_AW_: Airway pressure
pCO_2_: Arterial carbon dioxide partial pressure
PEEP: Positive endexpiratory pressure
P_plat_: Plateau pressure
RR: Respiratory rate
SrO_2_: Renal oxygen saturation
V_t_: Tidal volume
